# Consensus about image quality assessment criteria of breast implants mammography using Delphi method with radiographers and radiologists

**DOI:** 10.1186/s13244-020-00860-z

**Published:** 2020-04-03

**Authors:** Cláudia Sá dos Reis, Isabelle Gremion, Nicole Richli Meystre

**Affiliations:** 1 School of Health Sciences (HESAV), University of Applied Sciences and Arts Western Switzerland (HES-SO), Av. de Beaumont 21, 1011 Lausanne, Switzerland; 2 Discipline of Medical Radiation Sciences, School of Molecular and Life Sciences, Curtin University, GPO Box U1987, Perth, Western Australia 6845 Australia; 3 CISP - Centro de Investigação em Saúde Pública, Escola Nacional de Saúde Pública, Universidade NOVA de Lisboa, Lisbon, Portugal

**Keywords:** Mammography practice, Technique, Eklund, Breast positioning, Protheses

## Abstract

**Aims:**

To identify image quality criteria that can be applied to assess breast implant (BI) mammograms according to radiologists and radiographers’ perspectives and to explore the level of agreement about criteria priority.

**Methods:**

A two-round Delphi method using a questionnaire was applied to identify the level of agreement between experts, asking them to rank each image criteria available for mammography according to 4 possible answers (1 = need to have, 2 = nice to have, 3 = not pertinent/appropriate, 4 = do not know). Criteria for craniocaudal (CC), mediolateral-oblique (MLO) and lateral (ML), with and without Eklund manoeuvre, were included. This process was repeated after removing the less relevant criteria.

**Results:**

Between first and second rounds, different results were obtained regarding the criteria to assess CC and MLO images. Details for anatomic areas were considered the most relevant by radiographers during the first round, while general criteria were prioritised during the second round. Radiologists focused more on analysis of the spread of the breast tissue, if the breast was aligned with detector’s centre and level of contrast. The analysis of implant flow, the BI anterior edge and the maximum retropulsion of BI when Eklund manoeuvre is performed were the specific aspects of BI imaging considered as relevant for assessment.

**Conclusions:**

The importance of each criterion used to assess BI mammograms was not the same between radiographers and radiologists, suggesting the two groups of experts are looking for different requirements from the image. Further education and training is necessary to align strategies for assessing BI mammograms, and some criteria need to be adapted to reduce subjectivity.

## Key points.

- Two groups of experts (radiographers/radiologist) are looking for different IQ criteria.
- Eklund manoeuvre mammograms should have specific IQ criteria.
- BI mammograms must consider maximum retropulsion of the implant.
- Visualisation of the implant anterior edge means all breast tissue is included.
- Necessary to adapt the PNL criterion to different BI locations (subglandular/subpectoral).

## Introduction

Breast cancer screening programs (BCSP) are implemented across the world with the aim of reducing mortality by detecting cancer in its initial stage to increase chances of survival with earlier therapy [1]. Even being controversial [2], mammography is still considered the gold standard in some countries as the initial examination if equivocal clinical assessments or suspected implant complications are observed, particularly for women over 50 years old and those over 40 years with breast implants (BI) [3]. However, concerns about BI mammography have been raised due to the possible impairment of cancer detection [4–8]. Implants are denser compared to breast tissue [9] bringing challenges in image acquisition, reading and interpretation. The available guidelines for standard mammography do not present appropriate recommendations when a patient has BI, regarding protocols and techniques or how to evaluate image quality (IQ), namely which criteria should be considered to ensure that the exam is adequate to perform diagnosis on images with implants that are denser compared to breast tissue [10–13]. With the implementation of digital mammography and breast tomosynthesis in BCSP across Europe, it is important to establish what are the best approaches for imaging the breast, including those with implants, namely protocols and techniques, but also how to evaluate and interpret the images. Considering there is limited evidence in published literature about IQ criteria for BI mammography assessment [7, 9, 14–33], this study aimed to identify image quality criteria that can be applied to assess breast implant mammograms according to both radiologists’ and radiographers’ perspectives. It also aimed to explore the level of agreement regarding the priority of each criterion to distinguish between those that must be verified and those that are not a priority to determine when an examination needs to be rejected and/or repeated.

## Methods

To identify image quality criteria that can be applied to assess breast implant mammograms, a list of criteria available for standard mammography previously identified [34] was presented to a group of experts or stakeholders using a questionnaire and applying a two-round Delphi method [35–38]. The Delphi method provides an opportunity for experts/stakeholders to exchange viewpoints about a complex problem, to see how their evaluation of the issue aligns with others and to change their opinions, if desired, after reconsideration of the findings of the group’s work. The main stakeholders involved in this specific context are typically radiographers and radiologists. Radiographers have their role in the assessment of IQ immediately after acquiring the images and radiologists subsequently evaluate the images, to interpret and report the examination. Because of their respective roles, they were brought together and, with the guidance of a facilitator, their informed opinions were interrogated to create a final list of criteria to be used for assessing BI mammography examinations. The facilitator explained the objectives of this study and also the scale used to classify each criteria. The aim of the listed criteria was to help with deciding if an examination, including images in craniocaudal (CC), mediolateral-oblique (MLO), mediolateral (ML), acquired with and without Eklund Manoeuvre, presents all relevant imaging information to provide a diagnosis or if the examination should be repeated [39, 40]. The priority of each criterion was additionally explored. A consensus approach was used to define the level of agreement between the group members [35, 41]. According to the literature [35–37, 42], the group size can vary according to the purpose of the research or can be defined according to those who express interest in participating [36]. In this study, 10 participants (6 radiographers and 4 radiologists), all working in Swiss BCSP institutions, were invited to join the study after expressing their interest in the experience. All participants had a minimum of 7 years of experience in their respective profession.

The classical Delphi method was followed and involved 5 steps [36]:

1. A questionnaire was submitted online to the experts (participants), which presented a list of criteria that was based on a previous study [34]. They were asked to identify which items could be applicable to assess BI mammograms and add others that were not noted in the list (Tables 1 and 2);
2. The experts examined the criteria to categorise them as important or not and to ranked them according to the perceived level of importance as (a) need to have, (b) nice to have, (c) not pertinent/appropriate and (d) I do not know;
3. Findings were analysed and presented to provide feedback to both radiographers and radiologists;
4. After subsequent readjustments, a second list of criteria was submitted for another round of assessment by the experts;
5. Finally, an agreement based on both rankings was attempted, to set a list of criteria to be applied by professionals working in a regional BCSP.

**Table 1:** Criteria to assess mammography examinations grouped by type (positioning, artefacts, sharpness, parameters) in craniocaudal, mediolateral oblique and mediolateral mammograms

Criteria	References	Type	1	2	3	4
Breast centrally placed	[13, 43–45]	Positioning (13)				
Presence of pectoral muscle (PM)	[11, 13, 45]					
Pectoral muscle visualised down to the level of PNL	[11, 13, 45, 46]					
Visualisation of retroglandular adipose tissue	[11, 13, 45]					
Medial border of the breast included on the image	[11, 13, 45, 46]					
Axillary tail demonstrated	[11, 13, 45, 46]					
Superior breast edge included	[13]					
Inferior breast edge included	[45]					
Full visualisation of inferior breast tissue	[45]					
Inframammary angle clearly demonstrated	[11, 13, 45, 46]					
Nipple in profile or transected by skin	[11, 13, 45, 46]					
Nipple in the midline (+/− 10°)	[11, 45]					
Symmetrical mirror images R/L images	[11, 13, 45, 46]					
No skin folds	[13, 45, 46]	Artefacts (3)				
No artefacts	[45, 46]					
Skin edges visualised	[13]					
Spread of breast tissue to differentiate adipose from fibroglandular tissue	[43, 44]	Sharpness/compression (4)				
Sharpness of glandular tissue	[13, 45, 46]					
Sharpness of vascular structures	[13, 44]					
Visually sharp reproduction of skin structure (rosettes from pores)	[13]					
Good penetration of thicker areas without over penetration of thin areas	[45, 46]	Parameters (2)				
Appropriate contrast	[44–46]					

1, need to have; 2, nice to have; 3, not pertinent/appropriate; 4, I do not know

**Table 2:** Criteria designed to assess the implant imaging in craniocaudal, mediolateral oblique and mediolateral mammograms

Criteria	Type	1	2	3	4
Maximum “*retropulsion”* of breast implant	Implant assessment (4)				
If Eklund is applied—visibility of implant edge in the image					
Maximum implant visualisation					
Absence of artefacts (implant)					

Parallel to the Delphi rounds, the same criteria identified during the literature review were used on a set of 1207 images to verify if they were applicable or not to real clinical scenarios and see if it is possible to assess each criterion when BI mammograms are acquired [34].

The 4-point Likert-type scale was used to rank each image criteria; the agreement percentage was calculated for the four levels of all criteria. The Kendall’s *W* (also known as Kendall’s coefficient of concordance) was then used to identify the level of agreement amongst the raters. Kendall’s *W* ranges from 0 (no agreement) to 1 (complete agreement) [47]. The statistical analysis was performed using Statistical Package for the Social Sciences (SPSS) version 23 and Excel software. Subgroup analysis by profession was also performed.

Approval was obtained from participant stakeholders. All participants gave their written informed consent.

## Results

The first round response rate was 100% (*n* = 10) and for the second round was 90% (*n* = 9). The number of criteria ranked during the first round were in total 25, distributed between positioning, parameters, sharpness/compression and artefacts, and were adapted to each projection. Additionally, 4 criteria to assess the implant itself in a mammographic examination were considered (Table 2).

A. Criteria to assess image quality of CC projections performed with Eklund manoeuvre performed in women with BI

Criteria classified as “need to have” were sharpness of glandular tissue, absence of artefacts with a degree of consensus of 80%; spread of breast tissue to differentiate adipose from fibroglandular tissue with a degree of consensus of 70%; nipple in profile, maximum retropulsion of breast implant, breast aligned to the detector, absence of skin folders and adequate contrast between anatomical structures with a degree of consensus of 60%. The visualisation of the medial border, retroglandular adipose tissue and axillary tail were considered as “nice to have” structures with a degree of consensus of 60%. The sharp reproduction of skin structure (rosettes from pores) was considered as not necessary (Table 3).

**Table 3:** Criteria scoring for craniocaudal projection applying Eklund manoeuvre combining the opinions of both groups (radiologists and radiographers) and respective percentage (%)

	1st round *n* (%)				2nd round *n* (%)			
Criteria	Need to have	Nice to have	Not appropriated	Do not know	Need to have	Nice to have	Not appropriated	Do not know
Breast centrally placed	6 (60)	4 (40)			5 (56)	4 (44)		
1st round: Presence of pectoral muscle 2nd round: Visualisation of retroglandular adipose tissue	3 (30)	6 (60)	1 (10)		5 (56)	4 (44)		
Medial border of the breast included on the image	5 (50)	4 (40)	1 (10)		6 (67)	3 (33)		
Axillary tail demonstrated	3 (30)	3 (30)	4 (40)		2 (22)	2 (22)	5 (56)	
Nipple in the midline (+/− 10°)	2 (20))	7 (70)	1 (10)					
Nipple in profile or transected by skin	6 (60)	4 (40)			3 (33)	6 (67)		
No skin folds	5 (50)	4 (40)	1 (10)		4 (44)	5 (56)		
Skin edges visualised	2 (20)	4 (40)	1 (10)	3 (30)				
Spread of breast tissue to differentiate adipose for fibroglandular tissue	7 (70)	3 (30)			4 (44)	5 (56)		
Sharpness of vascular structures	2 (20)	3 (30)	3 (60)	2 (40)				
Sharpness of glandular tissue	8 (80)	2 (20)			7 (78)	2 (22)		
Visually sharp reproduction of skin structures (rosettes from pores)		4 (40)	5 (50)	1 (20)				
No artefacts	8 (80)	2 (20)			5 (56)	3 (33)	1 (11)	
Symmetrical mirror images	2 (20)	7 (70)	1 (10)		1 (11)	7 (78)	1 (11)	
Appropriate contrast					6 (67)	3 (33)		
Correct exposure					6 (67)	3 (33)		
Visibility of implant edge in the image	5 (50)	3 (30)	1 (10)	1 (20)	3 (33)	5 (56)		1 (11)
Maximum “retropulsion” of the implant	6 (60)	4 (40)			7 (78)	1 (11)	1 (11)	

The CC images acquired without Eklund technique had an extra criterion: the absence of flow in the implant area and symmetrical (in mirror) CC images.

Between the first and second Delphi rounds, it was possible to exclude some criteria (Table 4) due to poor scoring attributed to them, namely visualisation of pores, visualisation of the pectoral muscle and vascular anatomy. Contrast and adequate radiation penetration of the breast tissues were suggested as relevant in the first round outcomes and so were introduced into the second round and ranked high amongst the criteria.

B. Criteria to assess image quality of standard MLO projections performed in women with BI

**Table 4:** Criteria raking (mean values based in Kendall’s *W*) comparison between first and second Delphi rounds regarding craniocaudal images performed with Eklund manoeuvre combining the opinions of both groups (radiologists and radiographers)

Criteria for craniocaudal projection of breast implant mammograms with Eklund manoeuvre			
Criteria	1st round (average)	Criteria	2nd round (average)
Sharpness of breast tissue	5.5	Sharpness of breast tissue	5.4
Absence of artefacts	5.6	Maximum retropulsion of implant	5.8
Spread of breast tissue	6.2	Visualisation of medial breast tissue	6.1
Nipple in profile	6.8	Adequate image contrast	6.1
Maximum retropulsion of implant	6.9	Adequate image penetration	6.1
Breast aligned with the detector’s centre	7.1	Visualisation of retroglandular adipose tissue	6.8
Visualisation of medial breast tissue	8.1	Breast aligned with the detector’s centre	7.0
Absence of skin folders	8.1	Absence of artefacts	7.2
Visualisation of implant’s anterior edge	8.6	Absence of skin folders	7.6
Visualisation of retroglandular adipose tissue	9.0	Spread of breast tissue	7.7
Nipple angle +/− 10°	9.9	Nipple in profile	8.5
Images in mirror	10.1	Visualisation of implant’s anterior edge	9.1
Axillary tail visible	10.5	Images in mirror	10.3
Skin line visible	11.6	Axillary tail visible	11.3
Visualisation of vascular anatomy	11.8	Visualisation of pectoral muscle	Left out
Visualisation of pectoral muscle	13.5	Nipple angle +/− 10°	Left out
Skin line visible	14.0	Visualisation of vascular anatomy	Left out
Adequate image contrast	Not included	Visualisation of pores along the skin	Left out
Adequate image penetration	Not included	Skin line visible	Left out

The most important criteria for MLO images were as follows: absence of artefacts with a degree of consensus of 78%; sharpness and spread of breast tissues, inframammary angle clearly demonstrated, full visualisation of inferior breast tissue and maximum visualisation of pectoral muscle, nipple on profile, breast aligned with the detector centre, with a degree of consensus of 67%.

The sharp reproduction of skin structure (rosettes from pores) and skin line visualisation were considered as not necessary.

Between the first and second Delphi rounds, some criteria were excluded as observed above for CC images (Table 5). One of criterion that became less relevant in the second round for images with BI was the level of visualisation of the pectoral muscle, namely the pectoral to nipple line (PNL). That criterion was left out of the questionnaire for the second round.

C. Criteria to assess image quality of ML projections performed with Eklund manoeuvre performed in women with BI

**Table 5:** Criteria rakings (mean values based on Kendall’s *W*) comparison between first and second Delphi rounds regarding mediolateral oblique image manoeuvre combining the opinions of both groups (radiologists and radiographers)

Standard mediolateral oblique projection of breast implants			
Criteria	1st round (average)	Criteria	2nd round (average)
Absence of artefacts	5.81	Adequate image contrast	6.22
Visualisation of inferior breast tissue	7.69	Adequate image penetration	6.22
Inframammary angle open and visible	7.69	Sharpness of breast tissue	7.06
Level of visualisation of pectoral muscle (PNL)	7.75	Inframammary angle open and visible	7.11
Spread of breast tissue	7.75	Axillary tail visible	7.11
Nipple in profile	7.88	Visualisation of inferior breast tissue	7.28
Sharpness of breast tissue	8.19	Visualisation of superior breast tissue	7.33
No flow (implant)	8.50	Breast aligned with the detector’s centre	7.94
Breast aligned with the detector’s centre	8.69	Absence of artefacts	8.5
Axillary tail visible	8.69	Spread of breast tissue	8.78
Absence of skin folders	9.25	Nipple in profile	8.83
Visualisation at least half of the implant	9.44	Visualisation of retroglandular adipose tissue	8.94
Visualisation of superior breast tissue	9.56	Absence of skin folders	9.67
Images in mirror (symmetry)	9.69	Visualisation at least half of the implant	10
Visualisation of retroglandular adipose tissue	10.56	Images in mirror (symmetry)	12.11
Visualisation of vascular anatomy	14.00	Inferior level of pectoral muscle	12.89
Skin line visible	14.06	Level of visualisation of pectoral muscle (PNL)	Left out
Visualisation of pores along the skin	15.81	No flow (implant)	Left out
Inferior level of pectoral muscle	Left out	Visualisation of pores along the skin	Left out
Adequate image contrast	Left out	Visualisation of vascular anatomy	Left out
Adequate image penetration	Left out	Skin line visible	Left out

For ML images, the absence of artefacts, spread and sharp visualisation of breast tissues and breast aligned with the detector were considered as the most important parameters to be included in the analysis of ML images with a degree of consensus of 80%. The visualisation of superior and inferior breast tissues, the absence of skin folders and the maximum retropulsion of the implant to reduce the superimposition over breast tissue were other criteria highlighted as important with a degree of consensus of 60%. Adequate contrast and penetration were parameters not considered during the first Delphi round, but from the second round, these parameters were ranked in fifth and sixth positions (Tables 6 and 7).

**Table 6:** Criteria scoring for mediolateral projection using Eklund manoeuvre combining the opinions of both groups (radiologists and radiographers) and respective percentage (%)

Criteria	1st round *n* (%)				2nd round *n* (%)			
	Need to have	Nice to have	Not appropriated	Do not know	Need to have	Nice to have	Not appropriated	Do not know
Breast centrally placed	4 (80)	1 (20)			5 (56)	4 (44)		
1st round: Pectoral muscle (PM) visible down to the pectoral muscle 2nd round: PM visible until the upper edge of the implant	3 ((60)	2 (40)			2 (22)	3 (33)	2 (22)	2 (22)
Visualisation of retroglandular adipose tissue	2 (40)	3 (60)			4 (44)	5 (56)		
Superior breast edge included	3 (60)	2 (40)			6 (67)	3 (33)		
Full visualisation of inferior breast tissue	3 (60)	2 (40)			6 (67)	3 (33)		
Inframammary angle clearly demonstrated	1 (20)	3 (60)	1 (20)		6 (67)	3 (33)		
Nipple in profile or transected by skin	3 (60)	1 (20)			4 (44)	5 (56)		
No skin folds	3 (60)	2 (40)			3 (33)	6 (67)		
Skin edges visualised			3 (60)	2 (40)				
Spread of breast tissue to differentiate adipose for fibroglandular tissue	3 (60)	2 (40)			4 (44)	5 (56)		
Sharpness of vascular structures	1 (20)		3 (60)	1 (20)				
Sharpness of glandular tissue	4 (80)	1 (20)			6 (67)	3 (33)		
Visually sharp reproduction of skin structures (rosettes from pores)		2 (40)	3 (60)					
Visibility of implant edge in the image	1 (20)	4 (80)						
Maximum “retropulsion” of the implant	3 ((60)	2 (22)						
No artefacts	4 (80)	1 (20)			5 (56)	3 (33)	1 (11)	
Symmetrical mirror images	3 ((60)	2 (40)			1 (11)	7 (78)		1 (11)
Appropriate contrast					7 (78)	2 (22)

**Table 7:** Criteria rakings (mean values based on Kendall’s *W*) comparison between first and second Delphi rounds regarding mediolateral image manoeuvre combining the opinions of both groups (radiologists and radiographers)

Criteria for mediolateral projection of breast implants mammograms with Eklund manoeuvre			
Criteria	1st round (average)	Criteria	2nd round (average)
Sharpness of breast tissue	5.2	Sharpness of breast tissue	6.0
Absence of artefacts	5.4	Visualisation of inferior breast tissue	6.8
Breast aligned in the detector’s centre	5.5	Maximum retropulsion of implant	6.8
Visualisation of superior breast tissue	6.4	Superior edge 2 cm	7.4
Visualisation of inferior breast tissue	6.4	Adequate image contrast	7.4
Spread of breast tissue	6.4	Adequate image penetration	7.4
Maximum retropulsion of implant	6.6	Visualisation of superior breast tissue	8.3
Absence of skin folders	6.9	Breast aligned in the detector’s centre	8.3
Images in mirror	6.9	Nipple in profile	8.4
Visualisation of retroglandular adipose tissue	7.6	Absence of artefacts	9.0
Visualisation of implant’s anterior edge	9.3	Absence of skin folders	9.6
Inframammary angle open and visible	10.0	Spread of breast tissue	9.9
Visualisation of pectoral muscle anterior edge	12.4	Inframammary angle open and visible	10.0
Visualisation of vascular anatomy	12.7	Visualisation of retroglandular adipose tissue	10.8
Visualisation of pores along the skin	13.3	Images in mirror	12.1
Skin line visible	15.0	Visualisation of pectoral muscle anterior edge	12.2
Nipple in profile	Left out	Visualisation of implant’s anterior edge	12.7
Superior edge 2 cm	Left out	Visualisation of vascular anatomy	Left out
Adequate image contrast	Left out	Visualisation of pores along the skin	Left out
Adequate image penetration	Left out	Skin line visible	Left out

**Fig. 1:**
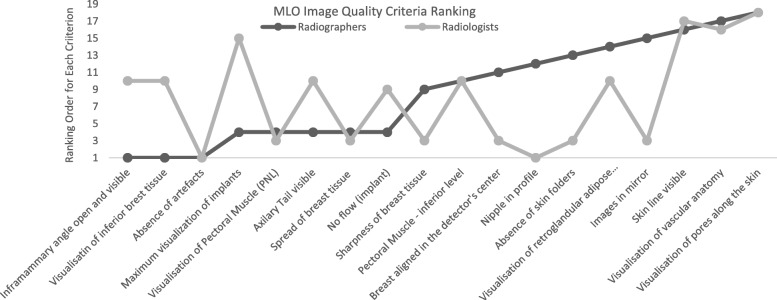
Rankings attributed to image quality criteria used to assess mediolateral oblique (MLO) images by radiographers and radiologists

**Fig. 2:**
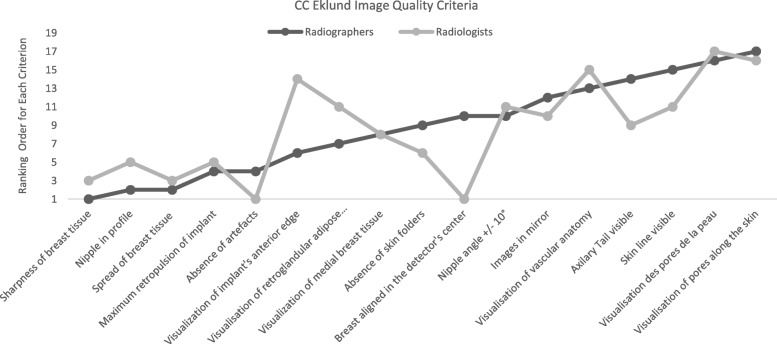
Average rankings attributed to image quality criteria used to assess craniocaudal (CC) images performed with Eklund Manoeuvre by radiographers and radiologists

Radiographers and radiologists did not agree in their ranking of 11 criteria for image quality assessment for the most common projections (CC and MLO) performed in women with BI (Figs. 1 and 2). The criteria considered as most relevant by radiographers to assess CC images were definition/sharpness of breast tissues, nipple in profile and spread of breast tissue. Radiologists noted the alignment of the breast with the detector’s centre as “need to have” as well as definition/sharpness of breast tissues. Radiographers considered it important in MLO views to visualise the inframammary angle and also the visibility of inferior tissues, while radiologists were looking for the absence of artefacts, nipple not superimposed over breast tissue, absence of skin folders and definition/sharpness of breast tissue.

Kendall’s coefficient of concordance was performed to verify the level of agreement between the two professional groups, and the differences were visible. The level of agreement between participants ranged from − 0.13 to 0.7 for craniocaudal image criteria and − 0.06 to 0.7 for MLO image criteria.

## Discussion

The objectives of this study were to identify image quality criteria that are currently in use to assess BI mammograms according to radiologists and radiographers’ perspectives and to explore the level of agreement about criteria priority.

To achieve these objectives a search was performed to identify possible criteria adapted to this specific context; however, no guidance was found [34]. This gap can impact on radiographers and radiologists’ activities considering that is important to know what should be demonstrated on the image to select the most suitable protocol and to achieve examination goals. Not having a level of image quality that allows for the analysis of all relevant anatomy of the breast with implants means the diagnosis of breast pathologies can be compromised [48].

This study showed that the two professional groups look at BI mammograms in different ways, having individual strategies to assess IQ as demonstrated by the results of the Kendall concordance test. The agreement between radiographers and radiologists ranged from weak (− 0.13) to good agreement (0.7). Major differences in agreement were related to the priority of criteria, with radiographers searching for specific anatomical details (nipple in profile, visualisation of medial, superior and inferior breast tissues), while radiologists were focused on overall assessment such as contrast, breast aligned with the detector, beam penetration, spread and sharpness of breast tissue and absence of artefacts. In a study promoted by the Canadian Association of Radiologists [49], the problems related to the presence of artefacts in the image were emphasised as they can promote an increase in false positive rates compromising the diagnosis. On the other hand, another study stressed positioning deficiencies as the main causes of inadequate image quality. The presence of skin folds, the pectoral muscle being concave or thin or having a sagging breast on the MLO, or a portion of breast cut off were frequently highlighted [50]. However, having a BI means the relevance and priority of some criteria can vary compared to standard mammograms. For example, the visualisation and shape of pectoral muscle will change if a subglandular implant is placed inside the breast because the implant will be overlapping with the muscle. A portion of breast being cut off can also happen in this situation, due to the limitations in manipulating the implant when it is encapsulated [51] or even to include inframammary angle in MLO projections [34].

This study also showed that the priority of each criterion is different amongst the two professional groups, it being desirable to take into account the likelihood of attaining each criterion in further studies. Radiographers prioritise the aesthetic side while radiologists look to see if the relevant information is noted in the image or not [52]. Previous studies showed that is effectively important as an overall assessment of image quality [43, 52], it being crucial to have all breast tissues included and correctly demonstrated. However, the proposed strategies to assess mammograms were still considered subjective for some criteria, and the need for standardisation was highlighted [53]. The words “appropriate” and “general amount” are being used but they are open to individual interpretation bringing variations in the final IQ analysis. Additionally, BI are an extra challenge because there is a superimposition of a dense structure over soft breast tissues, increasing the possibility of hiding relevant pathologies [43]. For that reason, modified positioning is required (Eklund) to help with the reduction of the amount of breast tissue superimposition. This is managed by displacing the implant posteriorly against the chest wall and pulling breast tissue over and in front of the implant, facilitating also the compression [51]. But these changes in positioning bring concomitant changes in the image appearance, making it necessary that radiographers know exactly what is required to be demonstrated, and communicating with radiologists to achieve a better alignment between both. Education also has a role to play as revealed in a previous study about mammography education in Europe [54] which demonstrated that positioning and image quality assessment are very challenging, leading to students demanding more training and a wider exposure to different clinical scenarios. Specific training for BI imaging could be an approach that would reduce professional differences.

The main limitations of this study are related to a different number of participants in both rounds (first 10, second 9) and also the 2 groups of professionals were not the same size (6 radiographers and 4 radiologists), which may affect the subgroup analyses. ML view and Eklund manoeuvre were not currently performed by all participants and implant location (subglandular or subpectoral) was not considered, and that has an impact on the visible anatomy as demonstrated previously [34]. This means that during the ranking process the decision of the participants would not consider the changes in image.

Further research is required for the identification of quality targets that should be reached in daily practice. However, the risk of omitting indicators was mitigated by the expertise of the panel who were given the opportunity to suggest additional ones, considering they were familiar with the relevant literature and BI imaging. That is also one of the advantages of using the Delphi method, where opinions can be different from one round to another [52], making the list richer.

Even with published work showing that is possible to use the Delphi method to identify quality indicators and prioritise criteria to be included in guidelines, it was challenging to conduct this study. The lack of standardisation of definitions, number of participants, optimal variance of rating scale, the best means for each answer and image quality assessment methodologies can lead to an incomplete list of criteria to assess BI examinations.

Therefore, basic criteria to start the image analysis were identified for BI such as maximum retropulsion of implant, visualisation of anterior edge of implant and no artefacts (such as flow). But some criteria identified as necessary are still subjective and that can be considered a limitation of this study, for example, “adequate contrast” and “adequate beam penetration”.

## Conclusions

Radiologists and radiographers did not consider the same parameters as relevant to assess image quality of BI mammograms; however, a list of criteria to assess BI mammograms was produced focusing on aspects of positioning, exposure parameters, sharpness and compression regarding the implant itself. This difference in the approach to image assessment shows that it is necessary to develop a standardised strategy in BI mammography, including different criteria adapted to each type of implant (subglandular versus subpectoral) as the changes promoted in the anatomy are different. Considering the experts’ opinions, the criteria to assess BI mammograms must consider maximum retropulsion of the implant, visualisation of the anterior edge of the implant and no artefacts (such as flow). The spread and sharpness of breast tissues are the other “need to have” parameters that do not differ from standard mammography. The revision of the PNL line and inclusion of the inframmamary angle criteria seem to be necessary to adapt to this specific context taking in consideration the implant location (subglandular/subpectoral).

Education and training to align radiographers and radiologists understandings is also necessary to have examination outcomes that match the interpretation requirements that lead to the optimal diagnostic outcomes of breast pathologies.

## Data Availability

Data generated or analysed during this study are included in this published article.

## Abbreviations

BCSP: Breast cancer screening programs
BI: Breast implants
CC: Craniocaudal
IQ: Image quality
ML: Mediolateral
MLO: Mediolateral oblique
PNL: Pectoral to nipple line
SPSS: Statistical Package for the Social Sciences
